# Identification and Analysis of Six Phosphorylation Sites Within the *Xenopus laevis* Linker Histone H1.0 C-Terminal Domain Indicate Distinct Effects on Nucleosome Structure

**DOI:** 10.1016/j.mcpro.2022.100250

**Published:** 2022-05-23

**Authors:** Fanfan Hao, Laxmi N. Mishra, Prasoon Jaya, Richard Jones, Jeffrey J. Hayes

**Affiliations:** 1 Department of Biochemistry and Biophysics, University of Rochester Medical Center, Rochester, New York, USA; 2 Department of Developmental and Molecular Biology, Albert Einstein College of Medicine, Bronx, New York, USA; 3 MS Bioworks, Ann Arbor, Michigan, USA

**Keywords:** H1 C-terminal domain, H1 phosphorylation, H1 CTD condensation, H1 phosphorylation affects nucleosome structure, mass spectrometry, FRET, nucleosome reconstitution, linker histone, nucleosome structure, linker DNA trajectory, CDK, cyclin-dependent kinase, CTD, C-terminal domain, FDR, false discovery rate, FRAP, fluorescence recovery after photobleaching, GD, globular domain, MS, mass spectrometry, MS/MS, tandem mass spectrometry, PTM, post-translational modification, TE, Tris and EDTA

## Abstract

As a key structural component of the chromatin of higher eukaryotes, linker histones (H1s) are involved in stabilizing the folding of extended nucleosome arrays into higher-order chromatin structures and function as a gene-specific regulator of transcription *in vivo*. The H1 C-terminal domain (CTD) is essential for high-affinity binding of linker histones to chromatin and stabilization of higher-order chromatin structure. Importantly, the H1 CTD is an intrinsically disordered domain that undergoes a drastic condensation upon binding to nucleosomes. Moreover, although phosphorylation is a prevalent post-translational modification within the H1 CTD, exactly where this modification is installed and how phosphorylation influences the structure of the H1 CTD remains unclear for many H1s. Using novel mass spectrometry techniques, we identified six phosphorylation sites within the CTD of the archetypal linker histone *Xenopus* H1.0. We then analyzed nucleosome-dependent CTD condensation and H1-dependent linker DNA organization for H1.0 in which the phosphorylated serine residues were replaced by glutamic acid residues (phosphomimics) in six independent mutants. We find that phosphomimetics at residues S117E, S155E, S181E, S188E, and S192E resulted in a significant reduction in nucleosome-bound H1.0 CTD condensation compared with unphosphorylated H1.0, whereas S130E did not alter CTD structure. Furthermore, we found distinct effects among the phosphomimetics on H1-dependent linker DNA trajectory, indicating unique mechanisms by which this modification can influence H1 CTD condensation. These results bring to light a novel role for linker histone phosphorylation in directly altering the structure of nucleosome-bound H1 and a potential novel mechanism for its effects on chromatin structure and function.

## Highlights

• Identified 10 sites of phosphorylation within the linker histone *Xenopus* H1.0.
• Six sites within the C-terminal domain *via* novel use of elastase as a protease.
• Individual phosphorylation indicates distinct effects on H1 CTD structure.
• Phosphorylation nearest to the GD directly regulates linker DNA trajectory.

## Graphical Abstract

**Figure:**
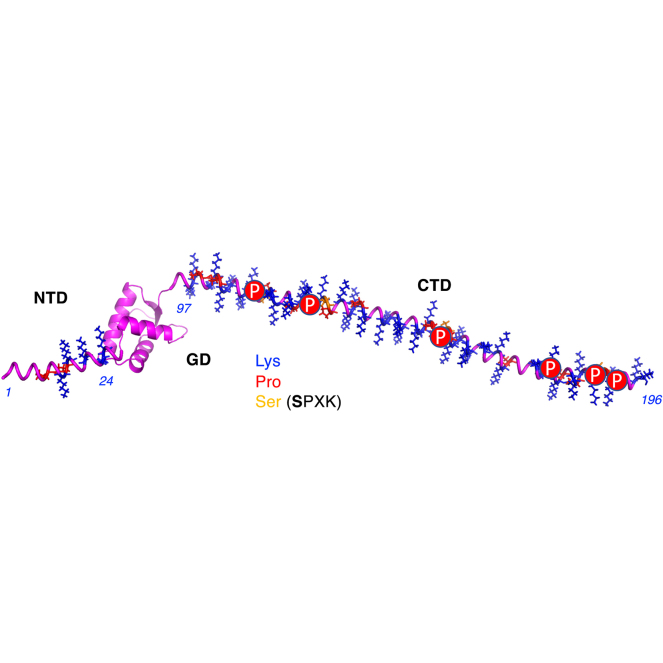

## In Brief

Linker histones are key components of chromosomes and phosphorylated in association with mitosis and other nuclear processes. However, the location of phosphorylation within the high-charged H1 C-terminal domain has been difficult to identify because of its repetitive and highly basic sequence. Moreover, the mechanism by which this post-translational modification alters nucleosome function remains unknown. We identify six sites of modification in the H1 C-terminal domain and demonstrate effects on H1 structure and H1-dependent nucleosome conformation.

The genomic DNA in eukaryotic cells is packaged into a hierarchical structure known as chromatin. The basic repeating subunit of chromatin is the nucleosome, which consists of 147 bp DNA wrapped around the core histone octamer, variable-length stretches of linker DNA, and, in the majority of cases, a single molecule of a linker histone (H1) (1). In higher eukaryotes, linker histones are nearly as abundant as the core histones and are essential for development of *Drosophila* and mouse embryos (1, 2, 3). Linker histones (H1s) can promote the folding and self-association of the extended arrays of nucleosomes into compacted higher-order chromatin structures, limiting the access of the nucleosomal DNA, though the exact organization of these structures is unclear and is the object of active investigations (4, 5, 6, 7).

H1s are comprised of three distinct domains, including a short N-terminal domain, a conserved central globular domain (GD), which directs the structure-specific binding of linker histones to nucleosomes, and a highly basic ∼100 amino acid residue C-terminal domain (CTD) (1). Most H1 CTDs contain ∼40 lysines roughly evenly dispersed along the ∼100 amino-acid residue long domain and are enriched in Ala, Pro, and Ser, and deficient in hydrophobic amino acids, consistent with the sequence content of an intrinsically disordered protein domain (8). In accord with this idea, the H1 CTD is unstructured in aqueous solution (9, 10); however, secondary structural elements have been detected in peptides derived from H1 CTDs upon interaction with DNA or in secondary structure–stabilizing solvents, such as 2,2,2 trifluoroethanol (11, 12, 13). Moreover, FTIR and NMR have detected signatures of secondary structural elements in the CTDs of H1 bound to chromatin (14, 15), and studies employing FRET monitoring the end-to-end distance across the CTD reveal a drastic condensation of this domain upon binding to nucleosomes consistent with a transition from a disordered state to ordered structure(s) (16, 17).

Linker histones facilitate the folding and compaction of long strings of nucleosomes into chromatin fibers and higher-order structures *in vitro* (18, 19). Importantly, the H1 CTD provides the majority of the overall binding free energy for H1 association in chromatin *in vivo* (20). Moreover, this domain is essential for H1’s chromatin-condensing function and for *Drosophila* development (19, 20, 21, 22, 23, 24). The H1 CTD structure is altered by the chromatin architectural factors HMGN1/2 (25), and epigenetic post-translational modifications (PTMs) within the H3 tail domain (26), suggesting this domain may be nexus for signaling in chromatin (27). Importantly, the extent of H1 CTD condensation is dependent on the earliest stages in folding of nucleosome arrays to condensed structures (28), raising the possibility that regulation of the propensity of the H1 CTD to undergo condensation may be a mechanism to fine-tune chromatin compaction.

Linker histones are phosphorylated by cyclin-dependent kinases (CDKs) in a cell cycle–dependent manner (29). Most of the CDK consensus sites (S/T)-P-X-(K/R) are localized in the CTD of linker histones (30). H1 phosphorylation levels are lowest in the G1 phase and increase during S and G2 phases, reaching peak levels in mitosis when chromosomes are maximally condensed (30, 31, 32, 33, 34). Studies suggest that threonine residues are mainly phosphorylated in mitosis, whereas CDK sites containing serine residues appear to be preferentially phosphorylated during both interphase and mitosis (31). H1 phosphorylation has been linked to chromosome condensation, as staurosporine, a CDK inhibitor, prevents H1 phosphorylation and cell entry in mitosis (35) and slows H1 exchange (36). Indeed, low levels of H1 phosphorylation in interphase have been linked to dynamic exchange of H1 and promoting chromatin decondensation (37). However, some studies suggest that histone H1 phosphorylation is dispensable for chromosome condensation (30). Using IR spectroscopy, Roque *et al.* (38) found that phosphorylation induced an increase in the proportion of β-sheet detected with a concomitant reduction in the amount of α-helix within the DNA-bound CTD peptides and full-length H1 in native chromatin *in situ* (14). Concomitant with linker histone partial phosphorylation, the sedimentation rate of chromatin decreased in a linear sucrose gradient, suggesting a relaxed and less compact chromatin structure (14).

Taken together, these data indicate that phosphorylation of H1 may influence chromatin folding by inducing alterations in H1 protein structure. However, the exact role of individual phosphorylation events in regulating H1 structure has not been extensively explored. Moreover, most phosphorylation sites in H1s, and especially in the CTD, have been predicted from knowledge of the CDK target site and have not been validated because of the repetitive nature of the H1 CTD amino acid sequence and the abundance of basic residues throughout this domain as the complete trypsin digestion results in small hydrophilic peptides, which are difficult to analyze by LC–MS/MS (39). Thus, we used novel mass spectrometry (MS) techniques to identify six sites of phosphorylation in the CTD of H1.0 isolated from circulating erythrocytes of *Xenopus laevis*. To investigate possible structural effects on the H1–nucleosome complex, we generated phosphomimetic mutants at the sites within the H1.0 CTD we identified *in vivo*. We found that all individual phosphorylation mimics, except for S130E, significantly altered the structure of the nucleosome-bound H1 CTD compared with the unphosphorylated H1. Furthermore, we found distinct effects among the mimetics on H1-dependent rearrangement of nucleosomal linker DNA, suggesting phosphorylation events affect H1 CTD condensation *via* multiple mechanisms.

## Experimental Procedures

### Linker Histone Preparation for *In Vitro* Studies

Linker histone H1.0b from *X. laevis* (here referred to as H1.0) and the phosphomimetic mutants were expressed in bacterial cells BL21(DE3) using the plasmid pET3aH1.0b (16). Briefly, 5 ml of an overnight culture was added to 500 ml LB containing 100 μg/ml ampicillin and incubated at 37 °C for about 2 to 3 h until the absorbance at 600 nm was 0.4 to 0.6. Protein expression was induced by the addition of IPTG (0.4 M) to a final concentration of 0.4 mM, and the culture was grown for another 4 h at 37 °C. Cell pellets were collected by centrifugation at 6000×*g* for 10 min at 4 °C, using a Sorvall RC6+ centrifuge and a Fiberlite F10 6x500y rotor, pellets dissolved in 20 ml TE buffer (10 mM Tris, 1 mM EDTA, pH 8.0), then 144 μl of 50 mg/ml lysozyme, 400 μl of 10% of Triton X-100, and 400 μl of 0.1 M DTT were added into the tube. Resuspended cells were incubated on ice for 30 min, and 5 M NaCl in TE was added to a final concentration of 1 M, then a cell lysate was obtained by sonication using a Branson 250 sonicator set to output 3 (duty cycle of 30%) for 1 min, three times each. After centrifugation at 26,000×*g* for 30 min at 4 °C, using Sorvall RC6+ centrifuge and Fiberlite F21s 8x50y rotor, the supernatant was recovered away from the cell debris (pellet) and NaCl was diluted to a final concentration of 0.75 M with TE. Three milliliters of a 50% suspension of Bio-Rex-70 50 to 100 mesh resin (Bio-Rad) was mixed with the supernatant, and the mixture was rotated for 1 h at 4 °C. NaCl concentration was diluted to 0.5 M with TE, and the mixture was rotated at 4 °C for another hour. Protein-bound resin was collected in a 25 ml disposable Poly-Prep chromatography column, washed twice with 20 ml 0.6 M NaCl (10 ml each) and 10 ml 0.7 M NaCl. H1.0 was eluted from the column with successive additions of 1 ml of 2 M NaCl, and eluates collected as 1 ml fractions, with a total elution volume of 20 ml. Fractions containing H1.0 were identified by running 10 μl of each fraction on SDS-PAGE. Peak fractions were collected together, then subjected to another round of purification as aforementioned except that 3 ml of a 50% slurry of Bio-Rex-70 100 to 200 mesh resin (Bio-Rad) was used. Nucleic acids were removed by incubation with 200 μl hydroxyapatite bead slurry (1:2 Bio-Rad hydroxyapatite: 1 M NaCl in TE) on ice for 1 h. H1.0 concentration was determined by quantitative comparison with an H1.0 standard determined by amino acid analysis.

### Core Histone Purification

The H3 used for this study contained a cysteine to alanine substitution at position 110. Histones H3 and H4 were expressed in bacterial cells BL21(DE3). Induction of expression of H3 and H4 was similar to that for H1 protein expression. Cells were lysed by sonication, centrifuged at 20,000×*g* for 30 min at 4 °C, the supernatant removed, and the pellets washed by resuspension in 10 ml 0.6 M NaCl/TE containing 10 mM DTT, followed by centrifugation, then the wash repeated with 10 ml of 1 M NaCl/TE containing 10 mM DTT, followed by centrifugation for 15 min at 13,000×*g*. This step was repeated with 10 ml 1 M NaCl containing 10 mM DTT. The pellet was resuspended in 5 ml 8 M urea and 2 M NaCl at room temperature with gentle mixing to liberate H3 or H4 from inclusion bodies. Nucleic acids were removed by adding 2 ml of a 50/50 hydroxyapatite bead slurry into the sample, rotating at 4 °C overnight, and beads removed by centrifugation at 5000×*g* for 5 min at 4 °C. After determining the relative concentration of proteins by SDS-PAGE, H3 and H4 were combined at a 1:1 molar ratio and dialyzed against 2 M NaCl/TE for 6 h, then dialyzed against 0.6 M NaCl/TE overnight. H3/H4 tetramer was purified by Bio-Rex chromatography as described previously (40, 41). Purified H3/H4 tetramer concentration was determined by quantitative comparison with standard H3/H4 tetramer.

Histones H2A and H2B were expressed in bacterial cells BL21(DE3). Expression of H2A and H2B was similar to that described previously for H1. After cell lysis and disruption by sonication, lysates were centrifuged at 13,000×*g* for 30 min at 4 °C. Pellets containing cell debris were discarded, and the relative concentration of H2A and H2B within supernatants was determined by SDS-PAGE gel. Supernatants containing equal molar amounts of H2A and H2B were combined, and the mixture was diluted to 0.5 M NaCl with TE. H2A/H2B dimer was purified with Bio-Rex 50 to 100 resin as described previously (41, 42).

### Preparation of 601 DNA Fragments for Nucleosome Reconstitution

DNA fragments for nucleosome reconstitution were generated by digestion of plasmid p207-12 with EcoRV. The 207 bp DNA fragments containing the 601-nucleosome positioning sequence were isolated from 0.8% agarose gels *via* electroelution.

### Nucleosome Reconstitution and Purification by Sucrose Gradient

Nucleosomes were reconstituted *via* a standard salt dialysis method (41). Briefly, 5 μg of H3/H4 tetramer, 5.8 μg H2A/H2B dimer, and 10 μg 601 DNA were mixed in reconstitution buffer (10 mM Tris, pH 8.0, 1 mM EDTA, 5 mM DTT, and 2 M NaCl) in a total volume of 300 μl, transferred to a dialysis tubing, then dialyzed against decreasing concentrations of NaCl (1.2, 1, 0.8, and 0.6 M), for 2 h at 4 °C, followed by dialysis against TE overnight at 4 °C. Reconstituted nucleosomes were purified on a 10 ml 7–20% sucrose/TE gradients by ultracentrifugation in a Beckmann SW41 rotor for 18 h at 34,000 rpm at 4 °C (supplemental Fig. S1). The fractions containing nucleosomes were pooled and concentrated using a microfuge tube filtration unit with a nominal molecular weight limit of 50 kDa (EMD Millipore). Nucleosome fractions were analyzed on a 0.7% native agarose gel and 18% SDS-PAGE.

### Attachment of Maleimide-Cy3 and Maleimide-Cy5 to Linker Histone

H1.0 G101C K195C, in which cysteines were located at either end of the H1 CTD, was incubated in 50 mM DTT for 1 h on ice to reduce the cysteines, then DTT was removed by Bio-Rex chromatography, and fractions were immediately frozen on dry ice. Fractions containing reduced H1.0 G101C K195C were treated with 5∼10-fold excess of either maleimide-Cy3, or maleimide-Cy5, or a 50/50 mix of both (GE Healthcare; catalog nos.: PA23031 and PA25031) for 30 min at room temperature in the dark. Free dyes were removed by another round of Bio-Rex cation-exchange chromatography. Concentration of fluorophore-labeled H1.0 was determined by quantitative comparison with an H1 standard, as described previously.

### FRET Analysis

Fluorophore-labeled H1.0 (final concentration of 5–15 nM) in H1 binding buffer (10 mM Tris–HCl, pH 8.0, 50 mM NaCl, 0.3% bovine serum albumin) was mixed with a range of amounts of purified mononucleosomes as indicated in the figure legends to ensure saturated H1 binding to nucleosomes. Emission spectra were recorded with excitation at 515 nm (Cy3 donor) and 610 nm (Cy5 acceptor) wavelengths with 5-nm slit widths in both excitation and emission channels on a Horiba Jobin Yvon FluoroMax-4 spectrofluorometer. The spectra of H1-binding buffer were also recorded and used for background subtraction. The ratio_(A)_ method was used to determine FRET efficiency (Equation 1) as described previously (43). The value (ratio)_A_ is the emission of acceptor excited at the donor excitation wavelength divided by the emission of acceptor under direct excitation (Equation 2).

(1) $$E=\frac{R_{0}^{6}}{R_{0}^{6}+R^{6}}$$

(2) $${\left(\text{ratio}\right)}_{\text{A}}=\frac{E{\text{ε}}^{\text{D}}\left({\text{λ}}^{\text{′}}\right){\text{d}}^{+}\;+{\text{ε}}^{\text{A}}\left({\text{λ}}^{\text{′}}\right)}{{\text{ε}}^{\text{A}}\left({\text{λ}}^{\text{″}}\right)}$$

In our experiment, (ratio)_A_ is measured by

(3) $${\left(\text{ratio}\right)}_{\text{A}}=\frac{F^{A}\left({\text{λ}}^{\text{′}}\right)}{F^{A}\left({\text{λ}}^{\text{″}}\right)}$$

Thus, FRET efficiency is:

(4) $$\frac{{\text{ε}}^{\text{A}}\left({\text{λ}}^{\text{″}}\right)\frac{F^{A}\left({\text{λ}}^{\text{′}}\right)}{F^{A}\left({\text{λ}}^{\text{″}}\right)}-{\text{ε}}^{\text{A}}\left({\text{λ}}^{\text{′}}\right)}{{\text{ε}}^{\text{D}}\left({\text{λ}}^{\text{′}}\right){\text{d}}^{+}}\;$$

*E* is the efficiency of energy transfer; *R* is the distance between the donor (Cy3) and acceptor (Cy5). *R*_0_ is the Förster distance that is dependent on the spectral overlap between donor and acceptor. ε^D^ (λ′) and ε^A^ (λ′) are the extinction coefficients of donor (Cy3) and acceptor (Cy5), respectively. d^+^ is the fraction of donor-labeled molecules, λ' is the wavelength for Cy3 excitation 515 nm, and λ'' is the wavelength for Cy5 excitation at 610 nm. Numerator represents the FRET intensity, and denominator represents the fluorescence signal from directly excited acceptor (Equation 2). F^A^(λ′) is the emission intensity of the acceptor (Cy5) fluorescence when exciting the sample at donor wavelength (515 nm); F^A^(λ'’) is the emission intensity of the acceptor (Cy5) fluorescence when exciting the sample at acceptor wavelength (610 nm). Note that (ratio)_A_ is independent of acceptor concentration. For this work, ε^D^(515) = 92,058 cm^−1^ M^−1^ (Cy3), ε^A^(515) = 6078 cm^−1^ M^−1^ (Cy5), and ε^A^(610) = 161,103 cm^−1^ M^−1^ (Cy5).

To eliminate issues with determination of d^+^ and absolute FRET efficiencies, herein, we report the FRET efficiency difference between H1 bound to nucleosome and H1 alone, Δ*E* (Equation 5), which can be derived from (Equations 1 and 2):

(5) $$\frac{\Delta E}{E_{\text{H}1\text{alone}}}=\frac{{\left(\text{ratio}\right)}_{\text{Aexp}}-{\left(\text{ratio}\right)}_{\text{AH}1\text{alone}}}{{\left(\text{ratio}\right)}_{\text{AH}1\text{alone}}-{\text{ε}}^{\text{A}}\left({\text{λ}}^{\text{′}}\right)/{\text{ε}}^{\text{A}}\left({\text{λ}}^{\text{″}}\right)}$$

Δ*E* is independent of d^+^, and only one accurate *E*_H1alone_ is required for the calculation of Δ*E*. Here, we determined d^+^ = 0.66 for labeled H1 alone as described before (43). All determinations are based on N ≥ 3 replicates. The linker DNA end-to-end distance FRET experiment was performed similar to the aforementioned H1 CTD FRET experiment. Note that fluorescently labeled nucleosome has Cy3 and Cy5 specifically incorporated at defined DNA ends, so d^+^ = 1. For this work, ε^D^(515) = 53,160 cm^−1^ M^−1^ (Cy3), ε^A^(515) = 3749 cm^−1^ M^−1^ (Cy5), and ε^A^(610) = 118,400 cm^−1^ M^−1^ (Cy5).

### Nuclei Isolation From *X. laevis* Blood

Blood from *X. laevis* was a kind gift from Prof Jacques Robert (University of Rochester, NY). Nuclei isolation from *Xenopus* blood was done as described previously (44). Briefly, blood was obtained from animals anesthetized with 0.25% MS-222 by heart puncture and collected into heparinized tubes. The blood was washed in modified PBS (1.79 mM KCl, 0.98 mM KH_2_PO4, 5.4 mM Na_2_HPO4, and 91.3 mM NaCl) three times (500*g*, 4 °C, 10 min) with the addition of 10 mM β-mercaptoethanol. The cell pellet was permeabilized in STKM buffer (250 mM sucrose, 50 mM Tris-HCl, pH 7.5, 25 mM KCl, 5 mM MgCl_2_, and 10 mM ß-mercaptoethanol) with 0.2% Triton X-100 followed by homogenization (five strokes) in a Dounce homogenizer. Homogenate was centrifuged at 1500×*g* for 10 min at 4 °C. The cytoplasmic fraction was discarded, and the pellet containing nuclei was washed two times with STKM buffer without any detergent.

### Isolation of Linker Histones From *X. laevis* Erythrocytes

Linker histones were extracted from the nuclei as described previously (45). Briefly, nuclei were resuspended in five volumes of 10% perchloric acid, and the mixture was incubated for 30 min on ice with occasional vortexing. After centrifugation at 14,000×*g* for 10 min at 4 °C, acid-soluble supernatant fraction containing linker histones was collected in a fresh tube. To precipitate proteins, trichloroacetic acid was added to a final concentration of 20%, mixed thoroughly, and incubated on ice for 1 h. The sample was centrifuged at 14,000×*g* for 15 min at 4 °C, the pellet was washed once with ice-cold acidified acetone (acetone + 0.1% HCl), once with ice-cold acetone (14,000×*g*, 10 min, 4 °C), and then air dried. The purified proteins were run on a 15% SDS-PAGE and stained with Coomassie blue. Two distinct bands were observed. Mass spectrometric analysis confirmed that upper band contained linker histones H1A, H1B, and H1C; whereas, the lower bands contained histone H1.0A and H1.0B.

### Enzymatic Digestion and MS

Lower bands containing H1.0 protein were washed in 100 mM NH_4_HCO_3_. DTT was added to a final concentration of 10 mM followed by incubation at 37 °C for 60 min. After that, iodoacetamide was added to alkylate proteins with final concentration of 20 mM followed by incubation at room temperature in dark for 30 min. We used either trypsin or elastase (Sigma) to digest the protein. Trypsin digestion resulted in poor coverage especially in the CTD as there are a number of lysines present, whereas elastase digestion yielded better coverage of the CTD region. Elastase was added to the H1 protein at 1:50 (w/w) for digestion at 37 °C overnight.

### Phosphoenrichment of Peptides

For phosphoenrichment, peptides were incubated with TiO2 microspheres for 30 min. The microspheres with enriched phosphopeptides were then collected by centrifugation, and the supernatant was removed. To remove nonspecifically adsorbed peptides, the microspheres were washed with 50% acetonitrile/6% TFA and 30% acetonitrile/0.1% TFA, sequentially. To elute the enriched phosphopeptides from the microspheres, elution buffer containing 10% NH_4_OH was added, and the enriched phosphopeptides were eluted using vibration. The supernatant containing phosphopeptides was collected and lyophilized for LC–MS/MS analysis.

### MS

Phosphoenriched (TiO_2_ treated) lyophilized peptides were dissolved in 0.1% formic acid and were directly loaded onto a reversed-phase precolumn (Acclaim PepMap 100; Thermo Fisher Scientific). Peptide separation was performed using a reversed-phase analytical column (Acclaim PepMap RSLC; Thermo Fisher Scientific) with a linear gradient of 6 to 22% solvent B for 22 min, 22 to 36% solvent B for 10 min, and 36 to 80% for 5 min at a constant flow rate of 280 nl/min on an EASY-nLC 1000 UPLC system. The resulting peptides were analyzed by Q Exactive hybrid quadrupole-Orbitrap mass spectrometer (Thermo Fisher Scientific).

The peptides were analyzed by tandem mass spectrometry (MS/MS) in a Q Exactive (Thermo) instrument coupled online to UPLC and nanospray ionization. Intact peptides were detected at a resolution of 70,000 in the Orbitrap and selected for MS/MS using 28% normalized collision energy. Ion fragments were detected in the Orbitrap at a resolution of 17,500. A data-dependent procedure that alternated between one MS scan followed by 20 MS/MS scans was applied for the top 20 precursor ions above a threshold ion count of 2E4 in the MS survey scan with 30.0 s dynamic exclusion with an applied electrospray voltage of 2.0 kV. Automatic gain control was used to prevent overfilling of the ion trap; 5E4 ions were accumulated for generation of MS/MS spectra. For MS scans, the *m/z* scan range was 350 to 1800.

### Database Search

The resulting MS/MS data were processed using Proteome Discoverer embedded (*version 1.3.0.339*) with Mascot search engine (*version 3.2*). Tandem mass spectra were searched against *protein sequence of Histone H1.0B* (*Xenopus*). Mass error was set to 10 ppm for precursor ions and 0.02 Da for fragment ions. Nonenzyme was selected. Carbamidomethylation on Cys was specified as fixed modification, oxidation on Met, and phosphorylation on Ser, Thr, and Tyr was set as variable modification. Peptide ion score was set to >13 (high confidence) with <5% false discovery rate (FDR) or ion score of <13 (low confidence) having FDR >5%.

### Experimental Design and Statistical Rationale

Individual FRET determinations to assess H1 CTD condensation were performed at least in triplicate, including fresh reconstitutions of nucleosomes. Determinations were performed at increasing amounts of nucleosome with a constant amount of H1 to ensure binding saturation of H1 with nucleosomes. Two-tailed Student’s *t* tests were performed to determine *p* values for pairwise comparisons. For linker DNA orientation experiments, a similar protocol was followed except that increasing amounts of H1 with constant amount of nucleosome were used.

## Results

**Fig. 1:**
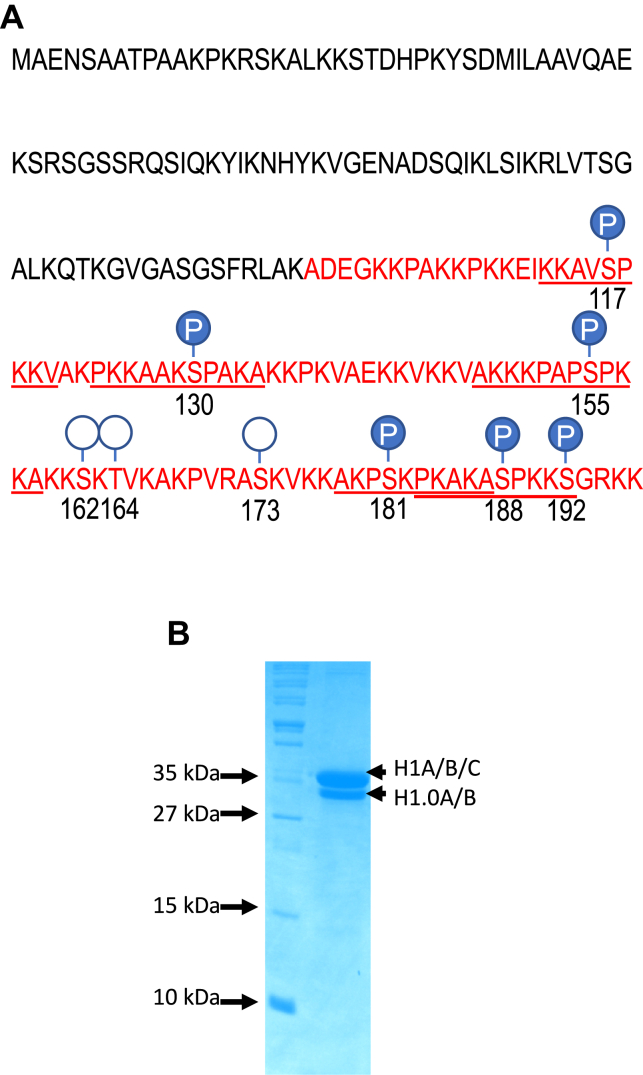
**Identification of phosphorylation with the *Xenopus laevis* H1.0 C-terminal domain (CTD).***A*, schematic showing predicted sites of phosphorylation within the H1.0 CTD (*circles*) and sites of *bona fide* phosphorylation identified in the current work (*filled circles*). *Underlines* indicate peptides used for identifications. *B*, H1 proteins purified from *Xenopus laevis* erythrocytes. Lane 1, molecular weight markers, lane 2, purified H1s. The H1 subtype content of the two major bands determined by mass spectrometry analysis is indicated.

We first aimed to identify sites of phosphorylation in the *X. laevis* linker histone H1.0, which is highly conserved between frogs and humans, and historically is one of the most studied linker histone variants. We first analyzed the protein sequence using Netphos 3.1, a widely used software package for phosphorylation site prediction (46, 47). In total, this software predicted nine sites of phosphorylation in the CTD with high confidence (Figs. 1*A* and S2*A*). Previously, it had been reported that S/TPXK motifs are sites of phosphorylation by CDK2 (31) in mammals. In order to identify the PTMs in endogenous H1.0, we isolated linker histones *via* perchloric acid extraction from *X. laevis* erythrocyte nuclei and observed two bands on SDS-PAGE (Fig. 1*B*). Both bands were digested with trypsin and subjected to MS/MS analysis (see Experimental Procedures section). We identified the upper band as containing H1 A/B/C and the lower band as consisting of H1.0 A/B. We subjected the lower band to a conventional trypsin digestion method followed by phosphoenrichment using TiO_2_. However, as expected, peptide coverage by this method was not satisfactory, covering only 49.5% of the protein sequence, with highest coverage in the N-terminal domain and GD and only 31.3% coverage of the CTD (supplemental Fig. S2*B*). We next employed elastase to digest the H1.0, as previously done for other linker histone digestions (48), as this protease cleaves peptide bonds after alanine, valine, glycine, leucine, isoleucine, and serine. Indeed, elastase digestion resulted in 71.9% protein coverage after mass spectrometric analysis, including 72.7% coverage of the CTD (supplemental Fig. S2*B*). Thus, elastase is superior to trypsin for PTM identification within the *Xenopus* H1.0 CTD.

**Fig. 2:**
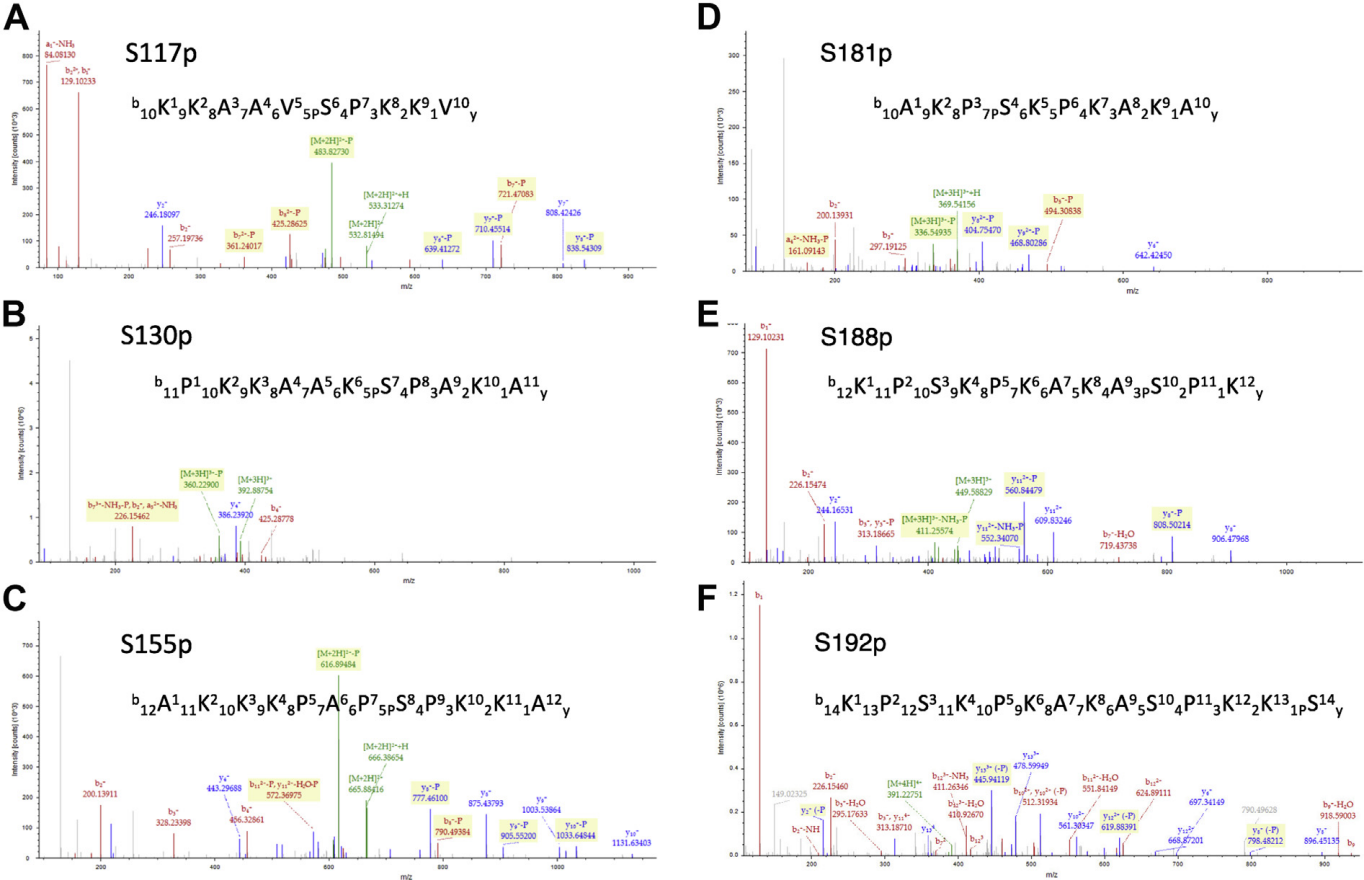
**Tandem MS (MS/MS) spectra of selected precursor ions identifying the indicated phosphorylations.***A*, precursor ion with an *m/z* value at 532.81^2+^ identifying S117p. *B*, precursor ion with an *m/z* value at 392.89^3+^ identifying S130p. *C*, precursor ion with an *m/z* value at 665.88^2+^ identifying S155p. *D*, precursor ion with an *m/z* value at 369.21^3+^ identifying S181p. *E*, precursor ion with an *m/z* value at 449.59^3+^ identifying S188p. *F*, precursor ion with an *m/z* value at 391.23^4+^ identifying S192p. See Table 1 for more information.

Based on our mass spectrometric analysis, we identified six serine residues within the H1.0 CTD (S117, S130, S155, S181, S188, and S192) as sites of phosphorylation *in vivo* (Figs. 1*A* and 2, and Table 1). (We also identified four sites of phosphorylation in the H1.0 GD but did not investigate these further; supplemental Fig. S3). Five of the CTD sites (S117p, S155p, S181p, 188p, and 192p) were identified with a high degree of confidence, with both phosphorylated precursor ions and several *b* and/or *y* ions indicating phosphorylation identified in each spectra (Mascot ion scores 42, 38, 13, 30, and 19, respectively, with FDR <5%) (Table 1). However, S130p was identified with lower confidence (Mascot ion score 10), with a *b* ion and parent ions indicating phosphorylation within the peptide. However, phosphorylation at S130 has been identified within the orthologous protein chicken H5, and the S130 site matches the CDK consensus (49, 50). Therefore, despite the lower confidence, we investigated potential effects of phosphorylation at all six sites.

**Table 1:** Data for phosphopeptides derived from H1.0 CTD shown in Figure 1

Phosphorylation site	Peptide	Phos	Charge	Mono *m/z*	MH+	Room temperature (min)	MIS	Ions matched	Conf
S117p	KKAVSpPKKV	S5	+2	532.81573	1064.62419	3.70	42	7/94	H
S130p	PKKAAKSpPAKA	S7	+3	392.89120	1176.65906	3.28	10	6/114	L
S155p	AKKKPAPSpPKKA	S8	+2	665.88403	1330.76079	3.60	38	7/126	H
S181p	AKPSpKPKAKA	S4	+3	369.21152	1105.62000	3.28	13	9/102	H
S188p	KPSKPKAKASPpK	S10	+3	449.59079	1346.75782	3.59	30	9/132	H
S192p	KPSKPKAKASPKKSp	S14	+4	391.22720	1561.88698	5.51	19	5/154	H

Abbreviations: Conf, confidence of identification (see *Experimental procedures* section); MIS, Mascot Ion Score.

**Fig. 3:**
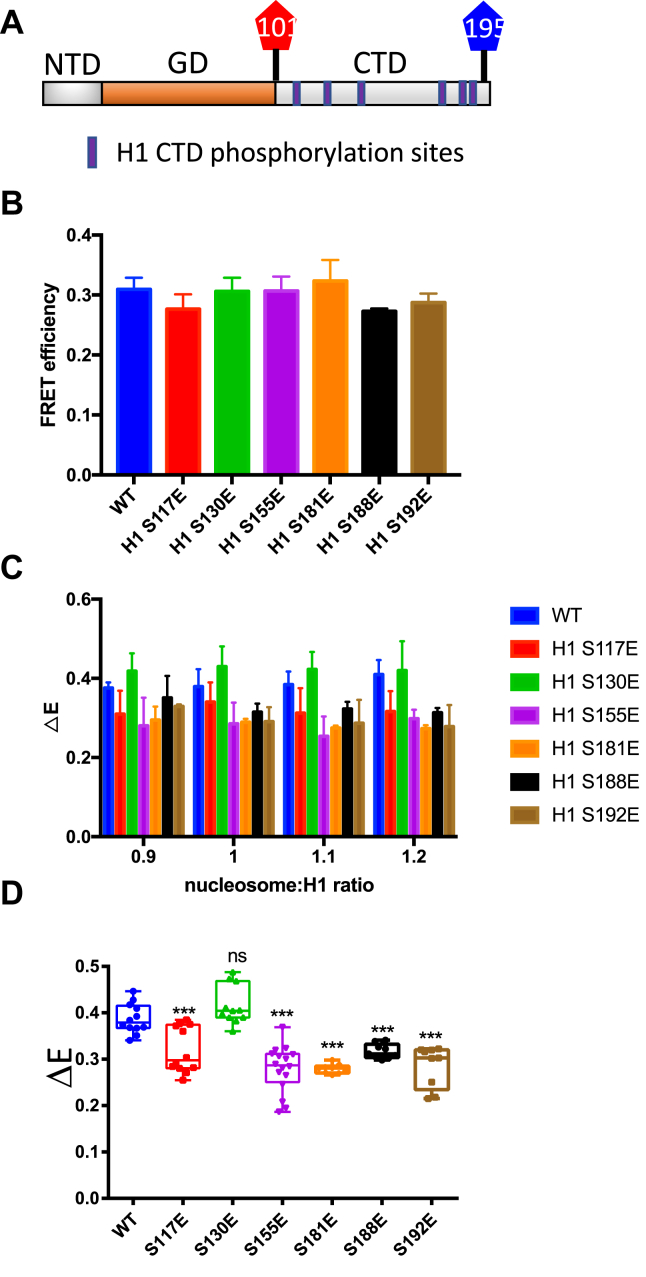
**H1 CTDs containing specific phosphomimetics exhibit distinct extents of condensation in nucleosome-bound H1s.***A*, schematic showing H1 G101C K195C labeled with the fluorophores Cy3 and Cy5 (*red* and *blue*) at residues 101 and 195 bracketing the H1.0 CTD. NTD, GD, and CTD denote the H1.0 N-terminal, globular, and C-terminal domains, respectively. Identified phosphorylation sites within the H1.0 CTD are indicated by the *thickvertical purple lines*. *B*, FRET efficiency of free H1 proteins. FRET efficiencies were determined for unmodified H1 and the H1 CTD phosphomimetic mutants as free proteins and plotted. *C*, graph of differences in FRET efficiency (Δ*E*) for samples with the indicated nucleosome:H1 ratios. Δ*E* was calculated as the difference between FRET efficiency for H1–nucleosome complexes and free H1. Error bars reported are SDs. N ≥ 3 for each determination. *D*, box plot of Δ*E* data shown in *C*. All data in which H1–nucleosome binding was saturated (ratios 1:1, 1:1.1, and 1:1.2) were combined and plotted, and *p* values were determined for the aggregate. *p* values indicate probabilities associated with two-tailed Student's *t* test. ∗∗∗*p* < 0.001. N ≥ 3. NS, not significantly different from WT. Colors are as in *C*.

We first investigated effects on nucleosome-induced CTD structure by individually substituting the six serines with glutamic acid to mimic phosphorylated serine within the context of H1.0 G101C K195C, in which two cysteine residues bracket the CTD, providing sites for attachment of fluorescent probes (Fig. 3*A*). The cysteines within the WT “unphosphorylated” H1.0 and the six H1.0 proteins bearing S → E phosphomimics were labeled with Cy3-and Cy5-maleimide, and FRET was used to assess the structure within the H1 CTD, either as free protein or upon binding to nucleosomes. Consistent with prior work, the free WT H1.0 exhibits a low FRET response, corresponding to an intrinsically disordered CTD (Fig. 3*B*). Analysis of the H1s containing the phosphomimetics indicated that the S → E substitutions do not alter the disordered state of the H1 CTD of the free proteins as the FRET efficiency of the free proteins was not significantly different from that of free WT H1 (Fig. 3*B*). The fluorophore-labeled H1s were then mixed with purified nucleosomes reconstituted with a 207 bp 601 DNA fragment and recombinant core histone proteins (supplemental Fig. S3) to determine if the phosphorylation mimics alter the characteristic nucleosome-induced CTD condensation (26).

We next measured the difference in FRET efficiency (Δ*E*) between nucleosome-bound and free labeled H1 in assays performed over a range of nucleosome:H1 concentrations. (Note in all cases, Δ*E* reaches a constant value at nucleosome:H1 ratios ∼1, indicating saturated [1:1] H1 binding to nucleosomes.) In agreement with previous work, Δ*E*, the difference in FRET efficiency between free and nucleosome-bound WT H1 was approximately 0.4, indicating that the intrinsically disordered CTD of WT H1 undergoes significant condensation upon binding to nucleosomes (Fig. 3*C*, *blue bars*). In contrast, all H1s bearing phosphomimetics except the S130E mutant exhibited a reduced ΔE compared with WT, between 0.28 and 0.32 (Fig. 3, *C* and *D*). These data indicate that phosphomimetics at S117, S155, S181, S188, and S192 caused a significant reduction in extent of H1 CTD condensation upon nucleosome binding compared with the unmodified WT H1. Interestingly, the S130E mimic had no effect on the extent of CTD condensation upon nucleosome binding, exhibiting a Δ*E* not significantly different than unmodified H1.

**Fig. 4:**
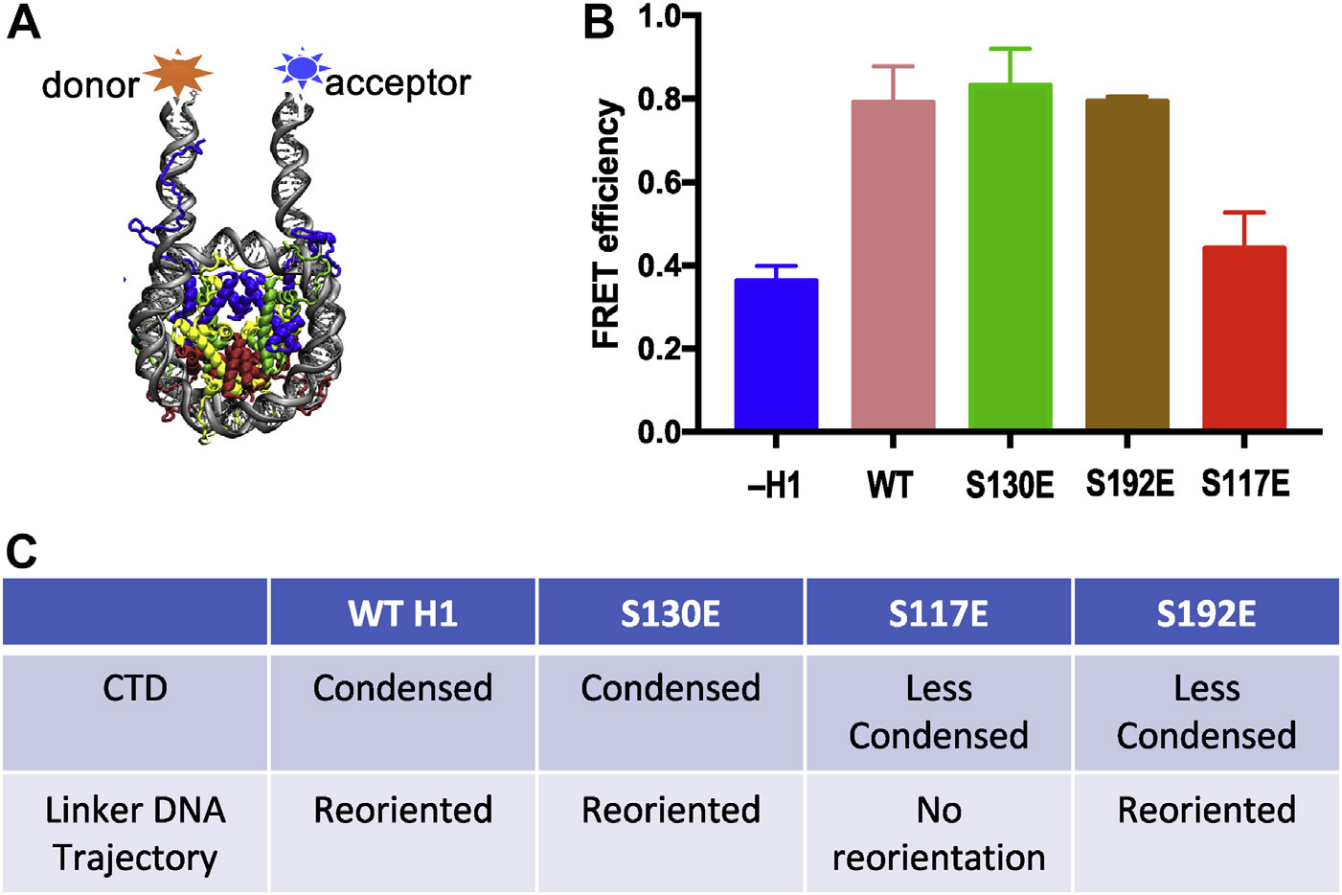
**Phosphomimetics in the H1.0 C-terminal domain (CTD) have distinct effects on linker DNA trajectory.***A*, schematic describing the fluorophore attachment sites near the linker DNA ends in the 207 bp nucleosome. *B*, FRET efficiency was determined for Cy3-and Cy5-labeled nucleosomes in the absence of H1 (–H1) or in the presence of the indicated H1. Error bars indicate standard deviation. *C*, summary of results of H1 CTD condensation and linker DNA reorientation assays.

We next set out to determine whether the phosphomimetics reorient linker DNA trajectory in the H1–nucleosome complex. Binding of H1 to nucleosomes reorients linker DNA by drawing the two linker DNAs close together, reducing end-to-end distance (51). Moreover, linker DNA trajectory is an important molecular determinant of H1 CTD condensation and potentially links CTD structure to folding/condensation of oligonucleosomes (28). We chose to investigate three of the phosphomimetic mutants, S117E, S130E, and S192E, which are positioned at the two ends and near the center of the CTD. Moreover, the S117E and S192E phosphomimetics induce a reduction in H1 CTD condensation upon nucleosome binding, whereas the S130E mimetic did not affect CTD structure as indicated by our FRET experiments. The ends of the DNA fragment used for nucleosome reconstitution were labeled with Cy3 and Cy5 donor and acceptor fluorophores, and FRET was used to determine the relative linker DNA end-to-end distance, which reflects the trajectories of the linker DNA segments emanating from the nucleosome core (Fig. 4*A*). In accord with our previous FRET data and cryo-EM structural analysis (26, 51), association of WT (unphosphorylated) H1 causes a large reduction in linker DNA end-to-end distance (Fig. 4*B*, *pink*). Likewise, the S130E and S192E mutants elicited a similar reduction in linker DNA end-to-end distance as WT H1 upon binding to nucleosomes (Fig. 4*B*, *green and brown bars*). In contrast, the S117E mutant drastically reduced the ability of H1 to reorient linker DNA (Fig. 4*B*, *red bar*). Of note, the effects of individual phosphorylation mimetics on linker DNA reorientation appear to be independent of effects on H1 CTD condensation (see Discussion section).

## Discussion

Using MS, we identified six novel phosphorylation sites within the CTD of *Xenopus* H1.0 with five being identified in our MS work with high confidence (S117p, S155p, S181p, S188p, and S192p) and one with lower confidence (S130p). We note, however, that S130p fits the CDK2 consensus site and was identified as a site of phosphorylation in the orthologous avian H5 (49). In order to determine how these PTMs might affect chromatin structure, we tested single phosphomimetics installed at each of these sites for effects on H1 CTD structure and H1-dependent reorganization of nucleosome linker DNA. We found that no single phosphomimetic resulted in detectable condensation or discernable change in structure of the intrinsically disordered CTD of free H1.0. However, upon binding to nucleosomes, all phosphomimetic mutants except S130E significantly reduced the extent of H1 CTD condensation upon nucleosome binding, compared with that observed for unmodified H1. Moreover, we find that H1 S117E reduces H1 CTD condensation and alters linker DNA conformation, whereas a phosphomimetic at S192 apparently alters H1 CTD conformation without altering H1-dependent linker DNA orientation.

H1 phosphorylation level is correlated with DNA aggregation capacity (38), and partial phosphorylation of H1 has been linked to chromatin decompaction and increased DNA accessibility (14). Traditionally, the H1 CTD has been proposed to function as an unstructured polycationic domain that is involved in chromatin condensation through charge neutralization (52, 53). Phosphorylation results in a decrease of the overall positive charge of H1 CTD because of the addition of a phosphate group, and so this modification might have been expected to either increase condensation of the disordered CTD in the free protein and/or enhance condensation in the nucleosome-bound H1. However, we find that none of the phosphomimetics causes a detectable condensation of the CTD in free H1.0 or an increase in condensation of the nucleosome-bound H1 CTD. Thus, our data suggest that a simple reduction in the overall positive charge within the H1 CTD is not a primary mechanism by which phosphorylation influences H1 CTD structure and point to distinct site-dependent effects by which this modification affects nucleosome-bound H1 CTD structure as well as the ability of H1 to define the trajectory of nucleosome linker DNA.

Our results are in agreement with fluorescence recovery after photobleaching (FRAP) studies showing that a single threonine to glutamic acid substitution at position 152 of human H1.1 caused increased H1 mobility, similar to that caused by truncation of the H1 CTD in which amino acid residues beyond position 151 were deleted (20). This result is difficult to reconcile by a simple unstructured polycationic domain model and suggests that the H1.1 T152E phosphomimetic destabilizes the H1–nucleosome interaction through an altered protein structure. Moreover, H1 phosphorylation has been shown to play a role in replication origin firing, and the recruitment of Cdc45, a factor required for initiation of replication and fork progression, correlates with H1 phosphorylation, likely by Cdk2 (54, 55). It should be noted, however, that we previously found that partial deletion of H1 CTD actually resulted in increased nucleosome-binding affinity, which is in contrast to the previous FRAP results (16). The discrepancy might be due to the fact that our H1–nucleosome binding experiment was carried out *in vitro* and the FRAP experiments were performed *in vivo*, where ancillary factors could contribute to the dynamics of H1. Indeed Pin1, a phosphorylation-specific prolyl isomerase, stimulated the dephosphorylation of H1 *in vitro* and modulated the structure of the CTD of H1 in live cells (56). Interestingly, about 70% of the Lys residues within the H1 CTD are present in doublets. In beta-sheet–like structure, the consecutive positive-charged side chains would project in opposite directions. This structural arrangement might be functionally relevant to the role that H1 plays in stabilizing the chromatin compaction (14).

We previously showed that linker DNA trajectory is an important molecular determinant of the extent of H1 CTD condensation in nucleosomes (28), and H1 binding to nucleosomes brings the two linker DNAs close together forming a stem structure (26, 57). We find that H1 S117E causes an increase in linker DNA end-to-end distance compared with that observed for unmodified WT H1 or the other phosphomimetics studied, suggesting that S117E affects H1 CTD condensation through altered linker DNA geometry. Interestingly, a seven amino acid region (121–127) of human H1.5 has been found to be essential for close apposition of the two linker DNAs and the formation of the stem structure in H1-bound nucleosomes (58). Though no phosphorylation site has yet been identified within this seven amino acid region in H1.5, it appears that phosphorylation of S117 in H1.0 may alter the ability of the nearby corresponding seven amino acid region in H1.0 to bring the two linker DNAs closer together and is reminiscent of the effect of phosphomimetics near a basic region in Tetrahymena H1 on transcription *in vivo* (59).

We find that phosphomimetics in the H1 CTD had distinct effects on H1 CTD condensation compared with linker DNA reorientation. For example, H1 S130E had no effect on nucleosome-induced H1 CTD condensation, resulting in a condensation commensurate to that of WT H1, and exhibited no effect on the ability of H1 to reorient linker (Fig. 4*C*). In contrast, S117E reduced nucleosome-induced H1 CTD condensation and also eliminated H1-dependent reorientation of linker DNA. Finally, S192E reduced nucleosome-induced H1 CTD condensation but has no effect on the ability of H1 to reorient linker DNA trajectory. Thus, an important conclusion from our work is that the effects of phosphorylation on the H1 CTD condensation and linker DNA structure are independent and distinct.

Recently, Fang *et al.* (28) found that the extent of H1 CTD condensation is dependent on chromatin conformation, specifically, the H1 CTD adopts more condensed structure when extended oligonucleosome arrays undergo salt-dependent folding. This coupling between chromatin compaction and H1 CTD structure presents an opportunity for regulation by PTMs that alter either CTD condensation and/or linker DNA trajectory. We identify several phosphorylation mimics that elicit either one or both of these effects, suggesting a function in regulating the stability of condensed chromatin. It will be interesting to extend our analysis to oligonucleosomes to investigate if specific H1 CTD phosphomimetics increase chromatin accessibility to transacting factors by promoting the opening of nucleosome arrays.

## Data Availability

All relevant data are within the article and its supplemental data files. The MS proteomics data have been deposited to the PRIDE repository with the dataset identifier PXD032292.

## Supplemental data

This article contains supplemental data.

## Conflict of interest

The authors declare no competing interests.
