# Supplementary material for: Identification and Analysis of Six Phosphorylation Sites Within the Xenopus laevis Linker Histone H1.0 C-Terminal Domain Indicate Distinct Effects on Nucleosome Structure
Source: Mol Cell Proteomics. 2022 May 23;21(7):100250. doi: 10.1016/j.mcpro.2022.100250 (PMC9243160; doi:10.1016/j.mcpro.2022.100250)
Supplement: Supplemental Fig. S1 [file mmc1.pdf]

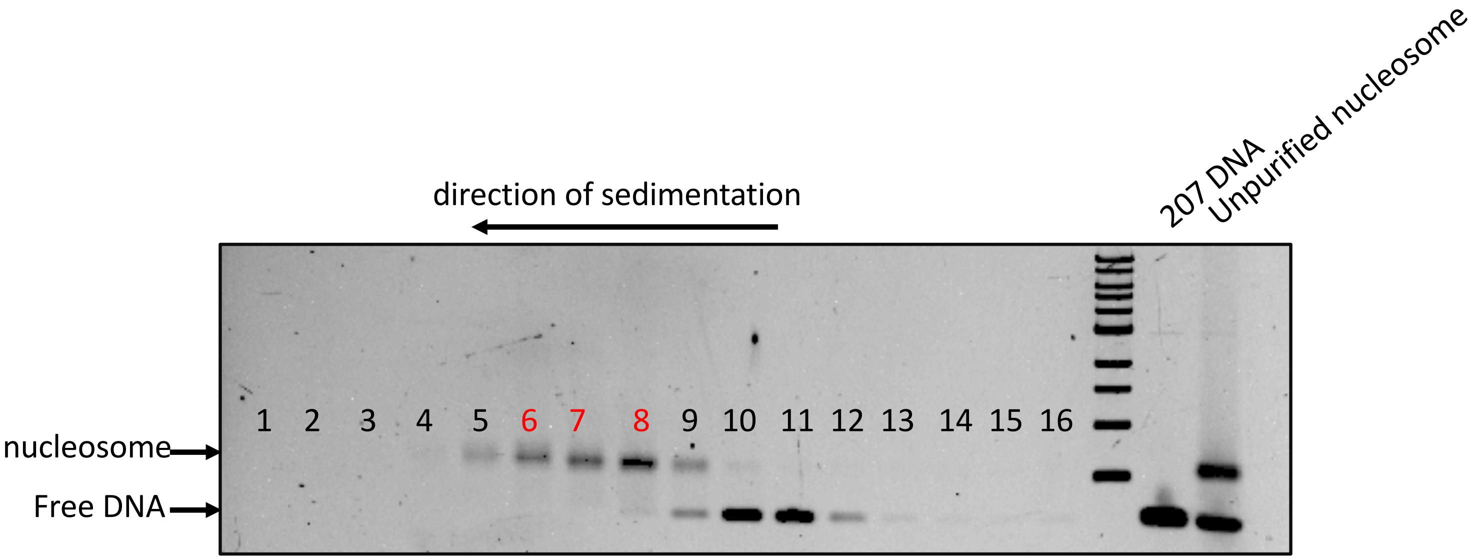

Fig S1. Preparation of nucleosomes reconstituted with Cy3 and Cy5 end-labeled DNA. Unpurified reconstitutions (last lane) were loaded onto a 7%-20% sucrose gradient, free DNA and nucleosomes separated by sedimentation as described in the Methods, and fractions analyzed by directly loading samples on 0.7% ½ X TBE gels. Fractions containing nucleosomes (fractions 6-8) were collected and used for further experiments. Arrow indicates the direction of sedimentation.
