# Supplementary material for: Identification and Analysis of Six Phosphorylation Sites Within the Xenopus laevis Linker Histone H1.0 C-Terminal Domain Indicate Distinct Effects on Nucleosome Structure
Source: Mol Cell Proteomics. 2022 May 23;21(7):100250. doi: 10.1016/j.mcpro.2022.100250 (PMC9243160; doi:10.1016/j.mcpro.2022.100250)
Supplement: Supplemental Fig. S2 [file mmc2.pdf]

Fig. S2

A.

Histone	Predicted sites of phosphorylation in CTD by Netphos3.1 software	Score (Phosphorylation by kinase)
Xenopus H1.0B	S117	0.997
	S130	0.992
	S155	0.998
	S162	0.795
	S164	0.94
	T173	0.939
	S181	0.931
	S188	0.996
	S192	0.963

B.

Trypsin digestion:

MAENSAATPAAKPKRSKALKKST
DHPKYSDMILAAVQAEKSRSGSSRQSIQKYIKNHYKVGENADSQIKLSIKRLVTSGALKQTKGVGASGSFRLAK
ADEGKKPAKKPKKEIKKAVSPKKVAKPKKAAKSPAKAKKPKVAEKKVKKVAKKKPAPSPKKAKKTKTVKAKPVR
ASKVKKAKPSKPKAKASPKKSGRKK

Peptides sequences covered by the trypsin digestion followed by LC-MS/MS is highlighted in yellow.
96 amino acids covered=48.97%
Total CTD covered=31 out of 99 amino acids=31.3%

Elastase digestion:

MAENSAATPAAKPKRSKALKKST
DHPKYSDMILAAVQAEKSRSGSSRQSIQKYIKNHYKVGENADSQIKLSIKRLVTSGALKQTKGVGASGSFRLAK
ADEGKKPAKKPKKEIKKAVSPKKVAKPKKAAKSPAKAKKPKVAEKKVKKVAKKKPAPSPKKAKKTKTVKAKPVR
ASKVKKAKPSKPKAKASPKKSGRKK
Peptides sequences covered by the elastase digestion followed by LC-MS/MS is highlighted in yellow.
141 amino acids covered= 71.94%
55 amino acids uncovered= 22.06

NTD:1-23: Red
GD: 24-97: Blue
CTD: 98-196: Black
Out of 99 amino acids in the CTD, 27 residues were not covered.
Total CTD covered= 99-27=72=72.72%

Fig. S2. A. Predicted phosphorylation sites in the CTD domain of Xenopus H1.0B using Netphos 3.1 software. B. Coverage obtained using trypsin and elastase digestion. The N-terminal, globular and C-terminal domains are indicated by green, black and red texts, respectively. Peptides identified by MS are highlighted in yellow.
