# Supplementary figures and images for: Identification and Analysis of Six Phosphorylation Sites Within the Xenopus laevis Linker Histone H1.0 C-Terminal Domain Indicate Distinct Effects on Nucleosome Structure
Source: Mol Cell Proteomics. 2022 May 23;21(7):100250. doi: 10.1016/j.mcpro.2022.100250 (PMC9243160; doi:10.1016/j.mcpro.2022.100250)

Fig. S3

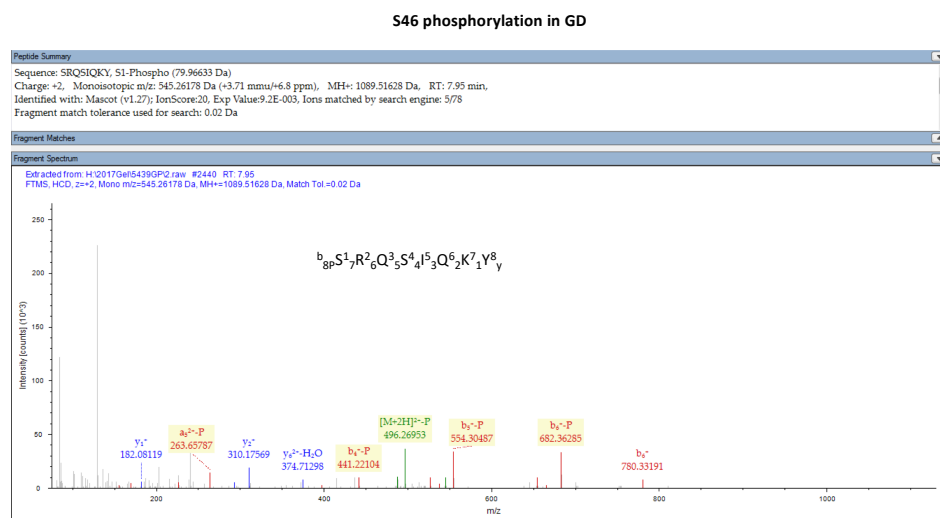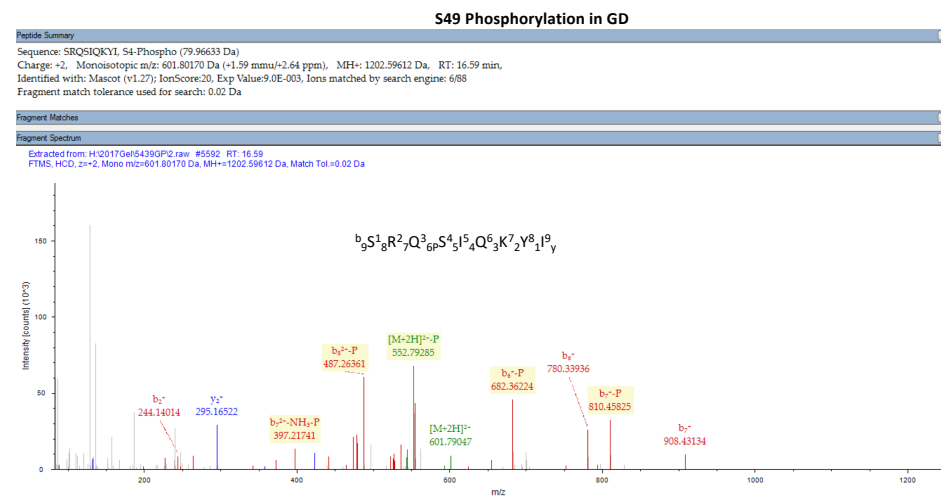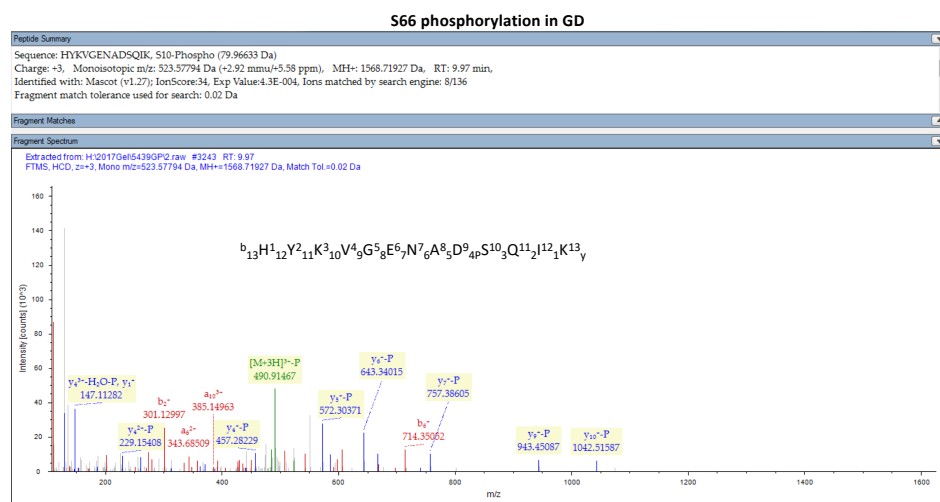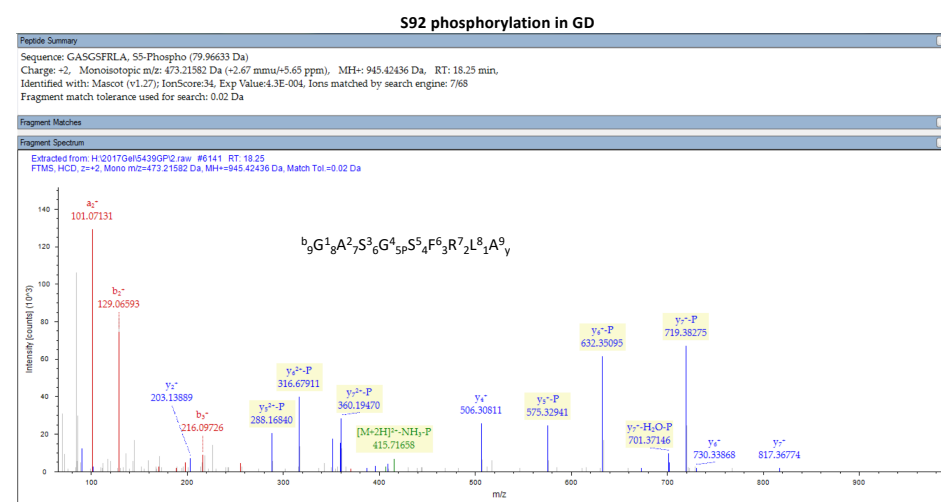

Fig. S2. Spectra of four sites of phosphorylation in the H1.0b globular domain

Supplement: Supplemental Fig. S3 [file mmc3.pdf]
